# Mapping and understanding of correlated electroencephalogram (EEG) responses to the newsvendor problem

**DOI:** 10.1038/s41598-022-17970-x

**Published:** 2022-08-13

**Authors:** Nghi Cong Dung Truong, Xinlong Wang, Hashini Wanniarachchi, Yan Lang, Sridhar Nerur, Kay-Yut Chen, Hanli Liu

**Affiliations:** 1 Department of Bioengineering, University of Texas at Arlington, 500 UTA Blvd, Arlington, TX 76019 USA; 2 Information Systems and Operations Management, University of Texas at Arlington, 701 S. Nedderman Drive, Arlington, TX 76019 USA; 3 Department of Business, State University of New York at Oneonta, 108 Ravine Parkway Oneonta, New York, NY 13820 USA

**Keywords:** Neuroscience, Environmental social sciences, Mathematics and computing

## Abstract

Decision-making is one of the most critical activities of human beings. To better understand the underlying neurocognitive mechanism while making decisions under an economic context, we designed a decision-making paradigm based on the newsvendor problem (NP) with two scenarios: low-profit margins as the more challenging scenario and high-profit margins as the less difficult one. The EEG signals were acquired from healthy humans while subjects were performing the task. We adopted the Correlated Component Analysis (CorrCA) method to identify linear combinations of EEG channels that maximize the correlation across subjects ($n=23$) or trials ($n=40$). The inter-subject or inter-trial correlation values (ISC or ITC) of the first three components were estimated to investigate the modulation of the task difficulty on subjects’ EEG signals and respective correlations. We also calculated the alpha- and beta-band power of the projection components obtained by the CorrCA to assess the brain responses across multiple task periods. Finally, the CorrCA forward models, which represent the scalp projections of the brain activities by the maximally correlated components, were further translated into source distributions of underlying cortical activity using the exact Low Resolution Electromagnetic Tomography Algorithm (eLORETA). Our results revealed strong and significant correlations in EEG signals among multiple subjects and trials during the more difficult decision-making task than the easier one. We also observed that the NP decision-making and feedback tasks desynchronized the normalized alpha and beta powers of the CorrCA components, reflecting the engagement state of subjects. Source localization results furthermore suggested several sources of neural activities during the NP decision-making process, including the dorsolateral prefrontal cortex, anterior PFC, orbitofrontal cortex, posterior cingulate cortex, and somatosensory association cortex.

## Introduction

Decision-making, one of the most critical activities of humans, is influenced by a complicated interplay among available information, external factors, and internal biases or constraints. Various studies on decision-making have focused on unveiling neurocognitive mechanisms while making decisions or identifying the engagement of different brain regions in such a process^1–5^. Functional magnetic resonance imaging (fMRI) has been the most popular brain imaging modality for determining different brain areas involved in the decision process^6–9^. However, although fMRI can provide high spatial resolution images of cerebral activities, its poor temporal resolution limits its application in studies requiring precise information regarding the relative timing of decision signals. Other imaging modalities with a better temporal resolution than that of fMRI, including electroencephalography (EEG)^10–12^ and magnetoencephalography (MEG)^13,14^, are employed to capture the fast neural responses underlying decision-making.

Many studies have utilized EEG concurrently with various decision-based tasks to investigate underlying neural mechanisms while making decisions^1,10,15–18^. Different EEG characteristics were analyzed to assess the decision-induced effects. For instance, Wyart et al.^1^ demonstrated that the delta band’s slow oscillations of EEG signals during categorical decisions were strongly correlated with the decision weight on a categorical choice. In^18^, Fink et al. investigated variations of EEG alpha power while subjects were making moving decisions in a soccer match. They observed substantial decreases in alpha power of parietal and occipital cortices in subjects making more creative moves. Golnar-Nik et al.^10^ extracted different features from EEG power and demonstrated that the customer’s decision to buy a product could be predicted using those features with high accuracy. Besides analyzing EEG oscillations of different frequency bands, many EEG studies on risk decision-making focused on analyzing event-related potentials (ERPs)^19–21^. Different ERP components such as Feedback Related Negativity (FRN), P3, N5, or N200^22–24^ were investigated to understand the neurocognitive mechanism underlying decision-making under risk. Other techniques, including Independent Component Analysis (ICA)^25^, Linear Discriminant Analysis (LDA)^11^, or Canonical Correlation Analysis (CCA)^26^, were also employed to extract event-related salient features. These features can be used to predict people’s future behavior or decisions based on the current outcomes^10,27^.

The newsvendor problem is one of the well-known problems in the field of operations management^28,29^ and behavioral research^30–33^. Such a problem deals with stocking level decisions in the presence of uncertainty for perishable goods (e.g., newspapers). For instance, if the newspaper vendors stock too much (overstocking) and are unable to sell all the stocked newspapers, they would have to incur a loss for the production/acquisition cost of the unsold newspaper. On the contrary, if the vendors stock too little (understocking), they will lose the opportunity to earn more profit. The newsvendor model has been applied to diverse decision-making contexts in manufacturing and service industries in operation management^34^. While traditional NP-based studies focused on deriving the optimal decisions to balance overstocking and understocking, recent research gained more interest in investigating human decision behavior in the newsvendor scenario^32,35^. However, most published papers on the NP were based on the behavioral analysis without quantitative interpretation of the mechanism of the newsvendor decision.

The goal of this study was to facilitate objective measure and evidence of the cognitive process during newsvendor decision-making by concurrently recording 64-channel EEG in response to the NP. Specifically, we designed an experiment in which two groups of participants faced the same dilemma as the NP, but at two difficulty levels for making decisions (and making money) when playing a computer game. Our hypotheses of the study included

(1) the attention state of the subjects is significantly correlated and modulated by the more difficult newsvendor decision across subjects and trials;

(2) the engagement or newsvendor-decision state of subjects modulates and reduces the alpha and beta-band power; and

(3) the neural activities during the NP process involve key cortical regions responsible for working memory, effortful attention, and computational and emotional processing.

**Figure 1:**
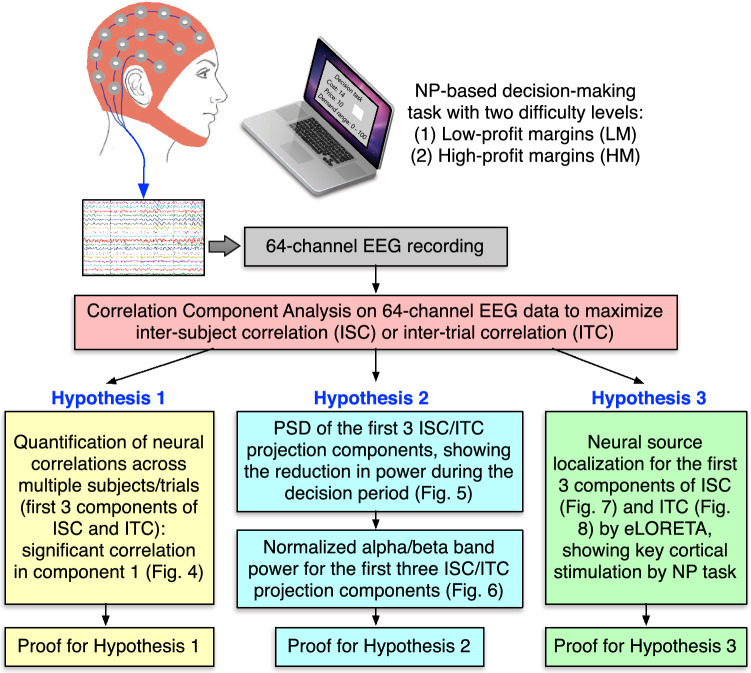
Flowchart outlining the hypotheses, procedural steps, and intended results for the study of 64-channel EEG data in response to the NP-based decision-making task.

By the end of this study, the experimental results would facilitate and support the hypotheses given above. Figure 1 shows a flowchart outlining the hypotheses, procedural steps, intended results, or conclusion.

## Materials and methods

### Participants, NP paradigm, and experimental setup

A total of 31 healthy subjects (mean age ± standard deviation (SD) of 26 ± 6.5 years) were recruited from the local community of The University of Texas at Arlington for this study. The data were collected only between 10:00 am and 5:00 pm. The experimental protocol was approved by the Institutional Review Board (IRB) of the University of Texas at Arlington. All methods were performed in accordance with the relevant guidelines and regulations. Informed consent was obtained from each participant prior to all measurements.

All subjects were healthy and did not have any neurological or psychiatric disorder history. Subjects were randomly assigned to two groups corresponding to two NP-based scenarios: low-profit margins (LM) and high-profit margins (HM). In the former, subjects found it hard to earn profit and frequently lose their money. Contrarily, in the HM context, subjects could easily gain substantial profit and rarely lost their money. The NP-based task protocol was explained to subjects prior to the experiment. Subjects could also try the game a few times (maximum 5) before starting the measurement. Participants were paid according to the equivalent accumulative profit they earned at the end of the experiment.

**Figure 2:**
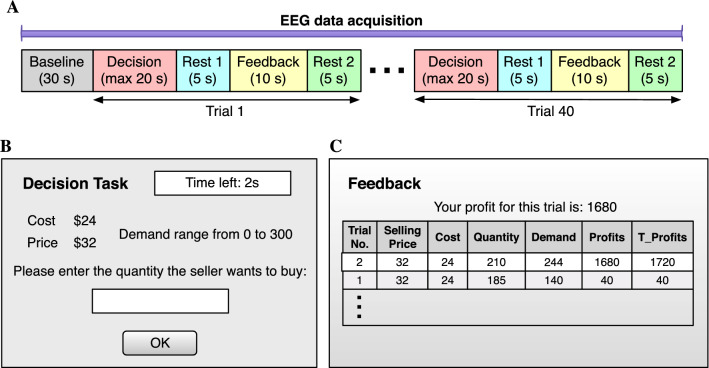
Experimental protocol. (**A**) Diagram of the NP-based experimental protocol. The whole experiment consisted of 30 s of rest (baseline) and 40 consecutive trials. Each trial included a maximum of 20 s of decision, 5 s of the first rest, 10 s of feedback, and 5 s of the second rest. EEG data were recorded throughout the whole experiment. (**B, C**) Computer screens of decision and feedback phases shown to subjects during the NP-based task.

The whole experiment for each subject consisted of 30 s of resting-state and 40 consecutive trials as shown in Fig. 2A. Each trial included a maximum of 20 s for the decision period, 5 s for resting before receiving 10 s feedback on the profit, and finally another 5 s of rest before starting the subsequent trial. The decision and feedback screens are depicted in Fig. 2B, C. During the decision period, subjects needed to decide the product quantity they wanted to buy based on the provided information, including the cost, the price, and the demand range of the product. The decision had to be made within 20 s. After 20 s, the product quantity would be set to 0 automatically, and the software switched to 5 s of the resting period. Feedback on the profit of the last trial was then displayed on the feedback screen for 10 s. EEG data were acquired throughout the whole experiment using a 64-channel EEG instrument (ActiveTwo, Biosemi, Netherlands). After carefully assessing the EEG data quality, we excluded 8 subjects due to the low signal-to-noise ratio, leaving 23 subjects, 12 for the LM and 11 for the HM groups.

### EEG signal preprocessing

64-channel EEG data were preprocessed using the EEGLAB toolbox^36^. The raw EEG signals were first bandpass filtered between 1-70 Hz to limit the bandwidth and further notch filtered at 60 Hz to remove line noise. Artifacts from eye blinks, eye movements, or jaw clenches were then removed using the Independent Component Analysis (ICA) method^37^. Finally, outlier EEG samples, whose magnitude exceeded four standard deviations of the channel’s mean, were replaced by zeros. Steps of the EEG preprocessing procedure are depicted in Figure SA.1A of Supplementary Material A.

### Correlation component analysis (CorrCA) across multiple subjects or trials

The first step in our EEG data analysis pipeline was to identify spatial combinations of EEG signals that maximize the correlation across multiple subjects or trials by using the CorrCA method developed by Dmochowski et al.^38,39^. The principle of CorrCA was similar to PCA, except that the components were computed to maximize the correlation of EEG data among multiple subjects or trials. CorrCA helped to convert EEG data from the electrode-by-time format to the component-by-time format, in which each component corresponds to an electrode combination. These components are sorted in descending order of across-subject or across-trial correlation. Since we kept only a few first correlated components, the number of components was much smaller than the number of electrodes, which helps mitigate the high-dimensionality problems of EEG data and makes further analysis of data more efficient.

**Figure 3:**
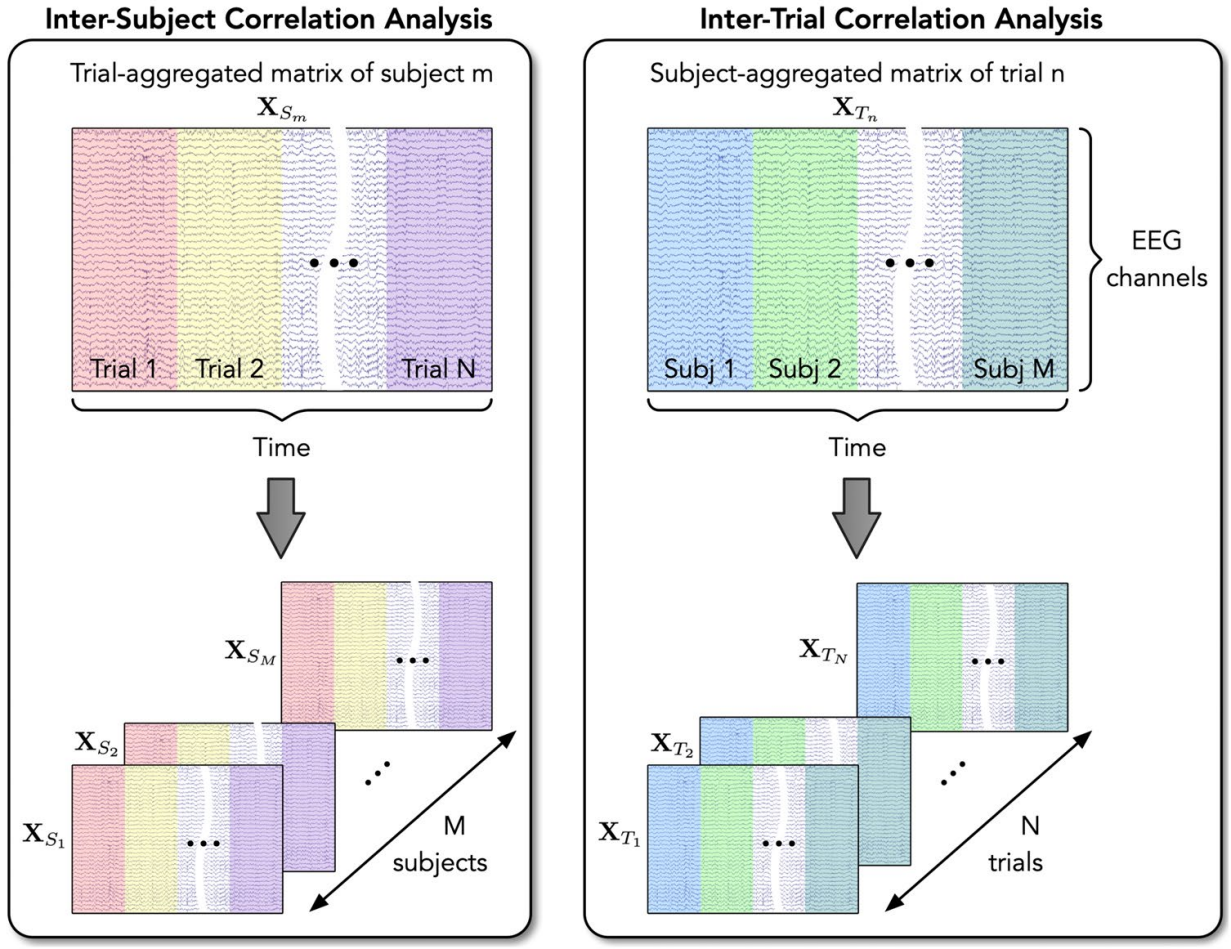
Illustration of the EEG data generation for the ISC and ITC analyses.

Given our specific experimental design, we opted for the CorrCA in two different data structures to maximize the inter-subject or inter-trial correlation (ISC or ITC). The EEG data generation for these two analyses was slightly different and is illustrated in Fig. 3.

For the ISC, we defined the trial-aggregated matrix $\mathbf{{X}}_{S_m}$ for subject *m* by concatenating multi-trial data along the temporal axis as follows:

1 $$\begin{aligned} {\mathbf{{X}}_{S_m}} = \left[ {\begin{array}{*{20}{c}} {{\mathbf{{X}}_{S_m,T_1}}}&{{\mathbf{{X}}_{S_m,T_2}}}&\cdots&{{\mathbf{{X}}_{S_m,T_N}}} \end{array}} \right] \end{aligned}$$

where ${{\mathbf{{X}}_{S_m,T_n}}}$ is the EEG data for trial *n* of subject *m* ($m=1\ldots M$ and $n=1\ldots N$, where *M* is the total number of subjects and *N* is the number of trials). ${{\mathbf{{X}}_{S_m,T_n}}}\in {\mathbb {R}}^{D\times N_T}$, where *D* is the number of EEG channels, and $N_T$ is the number of time samples of one trial.

Given *M* subjects participating in both LM and HM tasks, we had a set of *M* trial-aggregated EEG data $\left\{ \mathbf{{X}}_{S_1},\ldots ,\mathbf{{X}}_{S_M}\right\}$. The between-subject covariance $\mathbf{{C}}_b$ and the within-subject covariance $\mathbf{{C}}_w$ were calculated as follows:

2 $$\begin{aligned} \mathbf{{C}}_b= & {} \dfrac{1}{M(M-1)}\sum \limits _{i = 1}^M\sum \limits _{j=1,j \ne i}^M \mathbf{{C}}_{ij} \end{aligned}$$

3 $$\begin{aligned} \mathbf{{C}}_w= & {} \dfrac{1}{M}\sum \limits _{i = 1}^M \mathbf{{C}}_{ii} \end{aligned}$$

where $\mathbf{{C}}_{ij} = \text {cov}({\mathbf{{X}}_{S_i}},{\mathbf{{X}}_{S_j}})$ is the cross-covariance of EEG signals of subject *i* and subject *j* across all EEG channels, and $\mathbf{{C}}_{ii} = \text {cov}({\mathbf{{X}}_{S_i}},{\mathbf{{X}}_{S_i}})$ is the auto-covariance of EEG signals of subject *i*.

The linear combinations of EEG channels that maximize the correlation across multiple subjects were the solutions of the generalized eigenvalue problem:

4 $$\begin{aligned} \mathbf{{C}}_w^{-1} \mathbf{{C}}_b \mathbf{{w}} = \lambda \mathbf{{w}} \end{aligned}$$

We kept three solutions of equation (4), $\mathbf{{w}}_k$ ($k=1\ldots 3$), corresponding to the first three largest eigenvalues. The ISC $I_{m,k}^{\left<p\right>}$ of subject *m* and component *k* for period $\left<p\right>$, including ‘DCS’ for the decision period, ‘R1’ for the first rest, ‘FB’ for feedback, and ‘R2’ for the second rest, was estimated as follows:

5 $$\begin{aligned} I_{m,k}^{\left<p\right>} = \dfrac{\mathbf{w}_k^T \mathbf{{C}}_{b,m}^{\left<p\right>} \mathbf{w}_k}{\mathbf{w}_k^T \mathbf{{C}}_{w,m}^{\left<p\right>} \mathbf{w}_k} \end{aligned}$$

where the between-subject covariance matrix $\mathbf{{C}}_{b,m}^{\left<p\right>}$ and the within-subject covariance matrix $\mathbf{{C}}_{w,m}^{\left<p\right>}$ for subject *m* and experimental period ${\left<p\right>}$ were defined as:

6 $$\begin{aligned} \mathbf{{C}}_{b,m}^{\left<p\right>}= & {} \dfrac{1}{N}\dfrac{1}{M-1} \sum _{n=1}^N \sum _{i,i \ne m} \left( \mathbf{{C}}_{im}^{\left<p\right>_{T_n}} + \mathbf{{C}}_{mi}^{\left<p\right>_{T_n}}\right) \end{aligned}$$

7 $$\begin{aligned} \mathbf{{C}}_{w,m}^{\left<p\right>}= & {} \dfrac{1}{N}\dfrac{1}{M-1} \sum _{n=1}^N \sum _{i,i \ne m} \left( \mathbf{{C}}_{mm}^{\left<p\right>_{T_n}} + \mathbf{{C}}_{ii}^{\left<p\right>_{T_n}}\right) \end{aligned}$$

where $\mathbf{{C}}_{im}^{\left<p\right>_{T_n}}$ is the cross-covariance matrix computed from EEG data within period $\left<p\right>$ of trial $T_n$ for subjects *i* and *m*; $\mathbf{{C}}_{ii}^{\left<p\right>_{T_n}}$ is the auto-covariance matrix for subject *i* computed from EEG data within period $\left<p\right>$ of trial $T_n$.

For the case of computing the ITC, we generated the subject-aggregated matrix $\mathbf{{X}}_{T_n}$ for trial *n* by concatenating multi-subject data along the temporal axis as follows:

8 $$\begin{aligned} {\mathbf{{X}}_{T_n}} = \left[ {\begin{array}{*{20}{c}} {{\mathbf{{X}}_{S_1,T_n}}}&{{\mathbf{{X}}_{S_2,T_n}}}&\cdots&{{\mathbf{{X}}_{S_M,T_n}}} \end{array}} \right] \end{aligned}$$

The procedure for finding the linear combinations of EEG channels that maximize the correlation across multiple trials was similar to the process described above, except the input data, in this case, is a set of *N* subject-aggregated EEG data $\left\{ \mathbf{{X}}_{T_1},\ldots ,\mathbf{{X}}_{T_N}\right\}$, where *N* is the total number of trials of the whole experiment. The between-trial covariance $\mathbf{{C}}_b$ and within-trial covariance $\mathbf{{C}}_w$ were calculated using the cross- and auto-covariance of EEG signals of trials *i* and *j*, i.e., $\mathbf{{X}}_{T_i}$ and $\mathbf{{X}}_{T_j}$.

The CorrCA procedure was applied to all subjects to obtain the common linear combinations of EEG channels that maximize the correlation across multiple subjects or trials. The ISCs or ITCs of the first three CorrCA components were then calculated for each subject and for different experimental periods. Finally, in the statistical analysis step, we separated the ISCs and ITCs obtained from subjects participating in LM and HM tasks accordingly to investigate the effects of the task difficulty on the ISCs and ITCs of different experimental periods between these two groups. Figure SA.1B of Supplementary Material A outlines the steps for CorrCA across multiple subjects and trials.

### Normalized spectral power of spatially filtered EEG signals

We sought to analyze further the spectral power of spatially filtered EEG signals, namely, CorrCA projection components, to better understand cortical synchrony or desynchrony of brain responses evoked through different NP task periods. For example, desynchronization in the alpha band was reported being associated with increased attention^40^, while increased alpha oscillations were proposed to reflect an attention suppression mechanism^41^.

Given the linear combinations $\mathbf{{w}}_k$ ($k=1\ldots 3$) obtained from the generalized eigenvalue problem in equation (4), the projected components $\mathbf{{y}}_{k,S_m}$ of subject *m* is defined as follows:

9 $$\begin{aligned} \mathbf{{y}}_{k,S_m} = \mathbf{w}_k^T \mathbf{X}_{S_m} \end{aligned}$$

where $\mathbf{X}_{S_m}$ is the trial-aggregated EEG data of subject *m*, including the first 30 s of baseline. Note that although the linear combinations $\mathbf{{w}}_k$ can be obtained from ISC or ITC analysis, the time-resolved projection component $\mathbf{{y}}_{k,S_m}$ was calculated using the same EEG data for both cases.

For each projection component $\mathbf{{y}}_{k,S_m}$, we first computed the power spectral density (PSD) of every 1-sec data segment to generate a $[N_f \times N_t]$ time-resolved power spectrum, where $N_f$ is the number of frequencies, and $N_t$ is the whole task duration in second with the time resolution of 1 sec. The time-averaged power spectra of four periods of the NP task, including decision (DCS), first rest (R1), feedback (FB), and second rest (R2), were then calculated from all trials of each subject. We further computed the frequency-band-specific power of each task period for five commonly used EEG frequency bands, namely, delta (1–4 Hz), theta (4–8 Hz), alpha (8–13 Hz), beta (13–30 Hz), and gamma (30-60 Hz), by averaging the frequency-dependent power within the corresponding frequency range. The power during four task periods was also normalized by dividing by the frequency-band-specific baseline power. Statistical analysis was finally applied to investigate the difference in brain responses across four task/rest phases of two decision-difficulty levels. Figure SA.1C of Supplementary Material A outlines the steps for analysis of normalized power of CorrCA projection components.

### Sources of maximally correlated neural activity using exact Low Resolution Electromagnetic Tomography Algorithm (eLORETA)

As mentioned above, CorrCA led to multiple linear combinations of EEG electrodes that maximize the correlation of neural activity among different subjects or trials while performing the decision-making task. The “forward models”, which represent the scalp projections of the brain activities by the correlation components, were computed as follows:

10 $$\begin{aligned} \mathbf{{A}} = \mathbf{{C}}_w\mathbf{{W}}(\mathbf{{W}}^T\mathbf{{C}}_w\mathbf{{W}})^{-1} \end{aligned}$$

where $\mathbf{{C}}_w$ is the within-subject covariance matrix and $\mathbf {W}$ is a weight matrix whose columns are *K* largest eigenvectors with $\mathbf{{w}}_k$ obtained from equation (4). Each column of $\mathbf {A}$ corresponds to a forward model, which depicts an approximate spatial distribution of the neural sources.

To investigate further the sources of correlated neural activity defined by the correlation component, we employed the eLORETA^42,43^ to translate the obtained forward models into distributions of underlying cortical activity. Compared to standardized LORETA (sLORETA), eLORETA can provide exact localization of deeper sources with zero error despite the presence of both measurement and biological noise^44,45^. As reported in^45^, eLORETA produced more accurate localization of active sources compared to sLORETA.

### Statistical analysis

Two-way factorial ANOVA with NP-based task (LM vs. HM) as between-subjects factor and task period (DCS, R1, FB, and R2) as within-subjects factor was first used to investigate the statistical significance for both ISC/ITC and projection component’s normalized power. ANOVA assumptions, namely, the normality distribution of each group and the homogeneity of variances, were validated prior to performing the analysis. Respective results for the Shapiro-Wilk test for normality and the Levene or Bartlett Test of homogeneity of variances can be found in Supplementary Material B. Post-hoc pairwise comparisons with FDR adjustment for multiple comparisons were then performed to test significant differences between LM and HM tasks within each level of task periods, as well as pairwise comparisons of task periods within each task. We report FDR adjusted p-values for all analyses involving multiple comparisons.

For the case of normalized power, since no significant difference was found between LM and HM groups by the two-way factorial ANOVA, we pooled these two groups and performed one-way repeated-measures ANOVA on the pooled data to test the task period effects. We also verified the normality and homoscedasticity characteristics of the pooled data to ensure that ANOVA usage was appropriate. Post-hoc pairwise comparisons were finally carried out using Tukey adjustment for multiple comparisons to assess significant differences across 4 task phases (DCS, R1, FB, and R2).

### Ethical approval

The studies involving human participants were reviewed and approved by the Institutional Review Board of the University of Texas at Arlington, Arlington, TX, 76019 (IRB protocol #2016-0787, initially approved on June 26, 2017). The patients/participants provided their written informed consent to participate in this study.

## Experimental results

Participants in this experiment were randomly assigned to two groups corresponding to the LM and HM scenarios. During the experiment, each subject had an initial rest of 30 s before trying 40 consecutive trials. Each trial consisted of a maximum of 20 s for the decision period (DCS), 5 s for the first rest (R1), 10 s for the feedback period (FB), and finally another 5 s for the second rest (R2) before starting the ensuing trial. After the exclusion of 8 subjects’ data, we had a total of 12 and 11 subjects in the LM and HM group, respectively. The CorrCA method was applied to two data structures to maximize the correlation across multiple subjects or trials. We first calculated the ISC and ITC of the first three CorrCA components for different task periods.

### ISC and ITC modulated by the engagement of subjects

**Figure 4:**
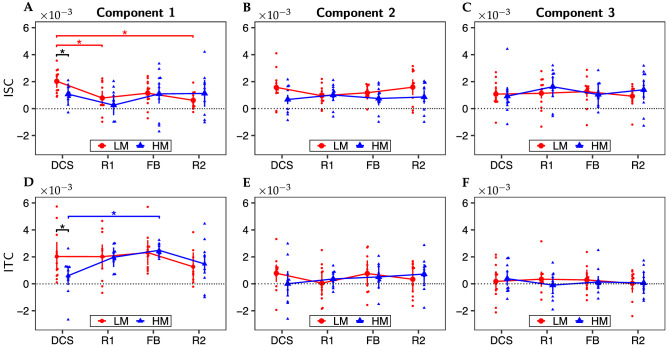
Results of the first three CorrCA components for (**A**–**C**) ISC and (**E**–**F**) ITC in response to LM (red; $n=12$) and HM (blue; $n=11$) decision-making tasks during four experimental periods, namely, decision (DCS), first rest (R1), feedback (FB), and second rest (R2). Significant differences between a period pair or between LM and HM tasks are marked as ‘*’ for $p< 0.05$ after FDR correction.

Figure 4 presents the ISC and ITC results of the first three CorrCA components of LM and HM tasks for four experimental periods. We employed two-way factorial ANOVA to investigate the statistical significance of ISC/ITC across NP-based tasks (LM and HM) and task periods (DCS, R1, FB, and R2). ANOVA assumptions, including the normality distribution of each group and the homogeneity of variances, were validated before performing the analysis. Respective results for the Shapiro-Wilk test for normality and the Levene or Bartlett Test of homogeneity of variances can be found in Supplementary Material B. Two-way factorial ANOVA revealed significant effects of task period factor for both ISC and ITC of the first component ($F = 4.25$, $p = 0.008$ for ISC and $F = 3.56$, $p = 0.019$ for ITC). No significant effects were found for the second and third components.

For the ISC analysis, follow-up pairwise analyses showed significant differences between DCS and R1 periods ($p_{FDR} = 0.024$, i.e., p-value after FDR correction for 12 comparisons) and between DCS and R2 periods ($p_{FDR} = 0.023$) for the LM task. Moreover, although two-way factorial ANOVA did not show a strong effect of the NP-based task factor ($F = 0.64$, $p = 0.43$), post-hoc analyses still revealed a significant difference between LM and HM tasks only for the DCS period ($p_{FDR} = 0.037$, FDR corrected for 4 comparisons). Overall, the ISC of the first component reached the maximum value during the DCS period for the more challenging task (i.e., the LM task). In other words, neural responses among subjects were strongly correlated by the attentional state, which is depicted clearly in Fig. 4A by the peak value during the DCS period of the LM task. In principle, the DCS period of the NP-based task would require the most engagement or attention of subjects compared to the other periods. While the HM task allowed the players to gain profit easily, the LM task would force them to engage and focus along with the protocol more promptly or concurrently to achieve profitable outcomes. Thus, ISC was significantly stronger during the DCS period for the LM task than the HM task and stronger than during two resting periods (R1 and R2) of the same LM task.

Post-hoc pairwise analyses for the ITC case showed a marginally significant difference ($p_{FDR} = 0.0496$, FDR corrected for 4 comparisons) between LM and HM tasks during DCS only, similar to the ISC case, as shown in Fig. 4D. In addition, another marginally significant difference was found between the DCS and FB periods for the HM task ($p_{FDR} = 0.048$, FDR corrected for 12 comparisons).

### Alpha- and beta-band power altered by the attentional state of subjects in different task periods

**Figure 5:**
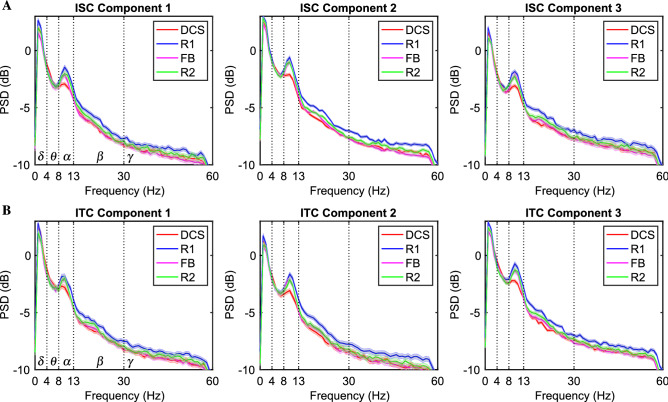
Averaged power spectral density (PSD) of 4 task periods (DCS (red), R1 (blue), FB (pink), and R2 (green)) for the first three ISC/ITC projection components calculated from all subjects of both LM and HM groups ($n=23$). Specifically, (**A**) depicts the PSDs of three ISC projection components, and (**B**) shows those of three ITC projection components. The shade of each curve indicated the standard error of the mean of each group.

As mentioned in the Materials and Methods section, we first calculated the time-resolved power spectra of the projection components. The time-averaged power spectra of 4 task periods were then computed by averaging the power over each task period duration and all trials for each subject. Figure 5 presents the averaged power spectral density (PSD) of 4 task periods for the first three ISC/ITC projection components. Note that the averaged PSDs were calculated from all subjects of both LM and HM groups ($n = 23$). For both ISC and ITC analyses, we observed the notable lowest spectral peaks of the DCS period within the alpha frequency band (red curves). In addition, the PSDs of the R1 period were almost consistently higher than those of other task periods within the alpha, beta, and gamma frequency bands.

**Figure 6:**
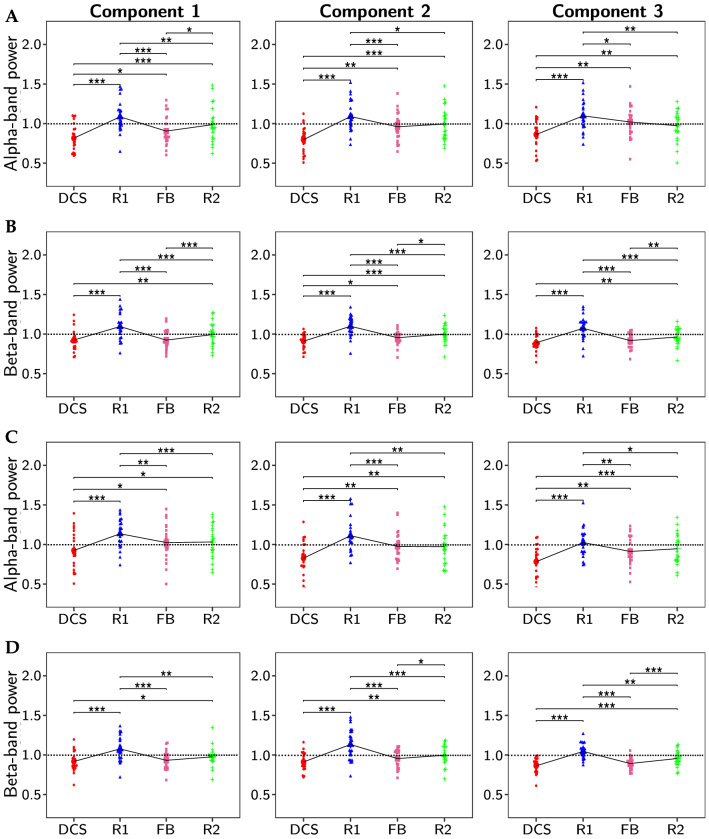
Normalized alpha- and beta-band power of the first three CorrCA projection components for ISC and ITC under four task periods (DCS, R1, FB, and R2) pooled from HM and LM. Specifically, (**A**) shows normalized alpha-band power of three ISC projection components; (**B**) normalized beta-band power of three ISC projection components; (**C**) normalized alpha-band power of three ITC projection components; (**D**) normalized beta-band power of three ITC projection components. Statistical results obtained by Tukey multiple comparison tests are mark as ‘***’ for $p_{\text {Tukey}} < 0.001$, ‘**’ for $p_{\text {Tukey}} < 0.01$, and ‘*’ for $p_{\text {Tukey}} < 0.05$.

We further calculated the frequency-band-specific power of each task period for each of the five EEG frequency bands and normalized it with the corresponding baseline power. As mentioned in the statistical analysis section, two-way factorial ANOVA with NP-based task as between-subjects factor and task period as within-subjects factor was first performed to investigate the statistical significance of normalized power of both factors. Since no significant difference in normalized power of the CorrCA projection components was found between the LM and HM groups by the two-way factorial ANOVA, we pooled these two groups and performed one-way repeated-measures ANOVA on the pooled data to assess the task period effects. Figure 6 depicts the normalized alpha- and beta-band power of the pooled data for the first three CorrCA projection components across 4 task periods: DCS, R1, FB, and R2. Figure 6A, B present power values of the components obtained by the ISC analysis, while Fig.  6C, D show those of the ITC analysis. ANOVA assumptions, namely, the normality distribution of each group and the homogeneity of variances, were also validated before performing the analysis. Respective validation results can be found in Supplementary Material B.

In the case of ISC, one-way repeated-measures ANOVA on the alpha-band normalized power indicated a strong effect of the experimental period factor for all three projection components (component 1: $F = 35$, $p =1.17 \times 10^{-13}$; component 2: $F = 26.4$, $p = 2.45 \times 10^{-11}$; component 3: $F = 19$, $p = 5.45 \times 10^{-9}$). Post-hoc pairwise comparisons with Tukey adjustment revealed significant differences for multiple period pairs, as shown in Fig. 6A. Similarly, one-way repeated-measures ANOVA on the beta-band normalized power of the ISC analysis also revealed significant differences among four task periods for all three projection components (component 1: $F = 36.7$, $p = 4.45 \times 10^{-14}$; component 2: $F = 37.3$, $p = 3.3 \times 10^{-14}$; component 3: $F = 39.9$, $p = 7.7 \times 10^{-15}$). Figure 6B show the results of Tukey multiple comparison tests among 4 experimental periods.

The alpha- and beta-band normalized power computed from the ITC spatial filters also led to results similar to those in the ISC case. Briefly, for the alpha-band normalized power, one-way repeated-measures ANOVA revealed significant differences among four task periods for all three components (component 1: $F = 14.9$, $p = 1.7 \times 10^{-7}$; component 2: $F = 22.7$, $p = 3.3 \times 10^{-10}$; component 3: $F = 18.6$, $p = 7.6 \times 10^{-9}$). Similarly, for the beta-band normalized power, the statistical results were as follows: $F = 28.1$, $p = 7.8 \times 10^{-12}$ for component 1, $F = 32.7$, $p = 4.5 \times 10^{-13}$ for component 2, and $F = 38.4$, $p = 1.8 \times 10^{-14}$ for component 3. The post-hoc analysis results are presented in Fig. 6C, D.

Figure 6 clearly shows overall similar trends in normalized powers at both alpha and beta bands for both ISC and ITC across four experimental periods. The normalized powers during the DCS phase were lower than 1 across all 12 cases, meaning that the power during the DCS period decreased compared to the baseline. On the contrary, the normalized powers during the R1 period were all higher than 1. For all cases, highly significant differences were found between DCS and R1 periods with $p_{Tukey} < 0.001$. Similarly, the normalized powers during FB were significantly lower than those during R1 in all 12 cases (i.e., at alpha- and beta-bands for both ISC and ITC). Thus, all these results demonstrate that the more the focus of subjects (i.e., during DCS and FB periods), the lower the alpha- and beta-band power. In other words, these results affirm that the engagement state of subjects modulated and reduced the alpha- and beta-band power.

For other frequency bands (Delta, Theta, and Gamma), statistical analysis did not reveal any significant differences across 4 experimental periods and 2 task types. Thus, no report for these frequency bands was presented.

### Sources of correlated neural activity for maximally correlated components using eLORETA

As mentioned in "Sources of maximally correlated neural activity using exact Low Resolution Electromagnetic Tomography Algorithm (eLORETA)" section, we would like to investigate further the brain sources that are responsible for the scalp projections of the brain activities by the maximally correlated components. eLORETA was employed to translate the ISC and ITC forward models into distributions of underlying cortical activity. The eLORETA-derived results suggest possible cortical origins of the neural activity while performing the tasks.

**Figure 7:**
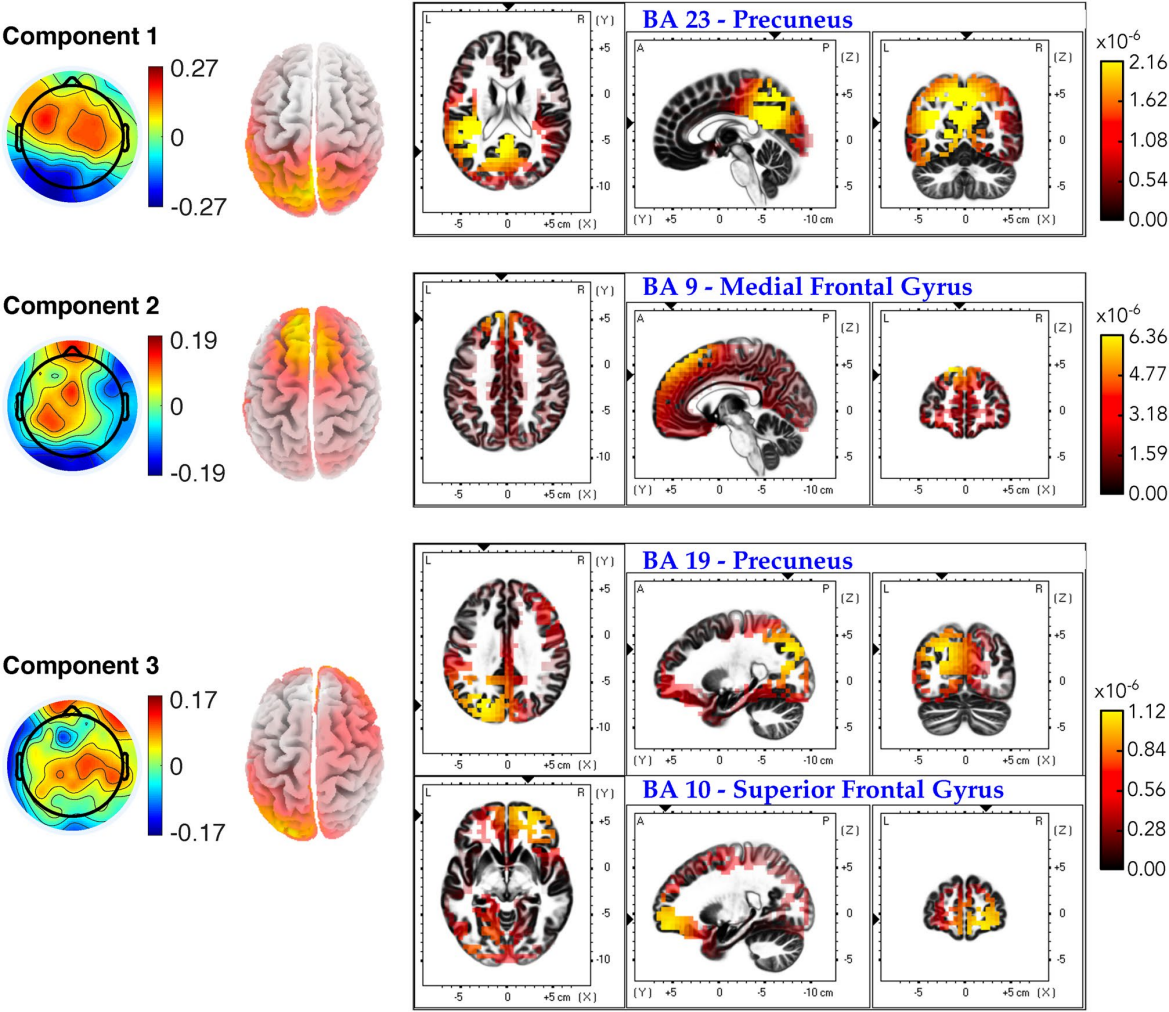
Neural source localization results for the first three ISC components. The scalp projections are shown in the leftmost column for respective components. The second column presents the estimated source distributions (top view). The remaining plots exhibit three orthogonal slices corresponding to the primary sources of the localization results. Full views of 3D source distributions for each component were depicted in Figure SC.1 of Supplementary Material C.

Figure 7 depicts the results obtained by using LORETA to estimate the neural source distributions from the scalp projections obtained by the ISC analysis. The localization results for the first component show one primary source in Brodmann Area (BA) 23 (Precuneus, Occipital Lobe), which is involved in memory and emotion^46^. The second component resulted in a significant source corresponding to BA 9 (Medial Frontal Gyrus, Frontal Lobe). BA 9 is believed to be involved in cognitive functions such as working memory, planning, and effortful attention^47^. Finally, the source analysis of the third component reveals possible sources from the BA 19 (Precuneus, Parietal Lobe) and BA 10 (Superior Frontal Gyrus, Frontal Lobe). While BA 19 is associated with complex processing of visual information, BA 10 is also involved in cognitive functions such as task management and planning.

**Figure 8:**
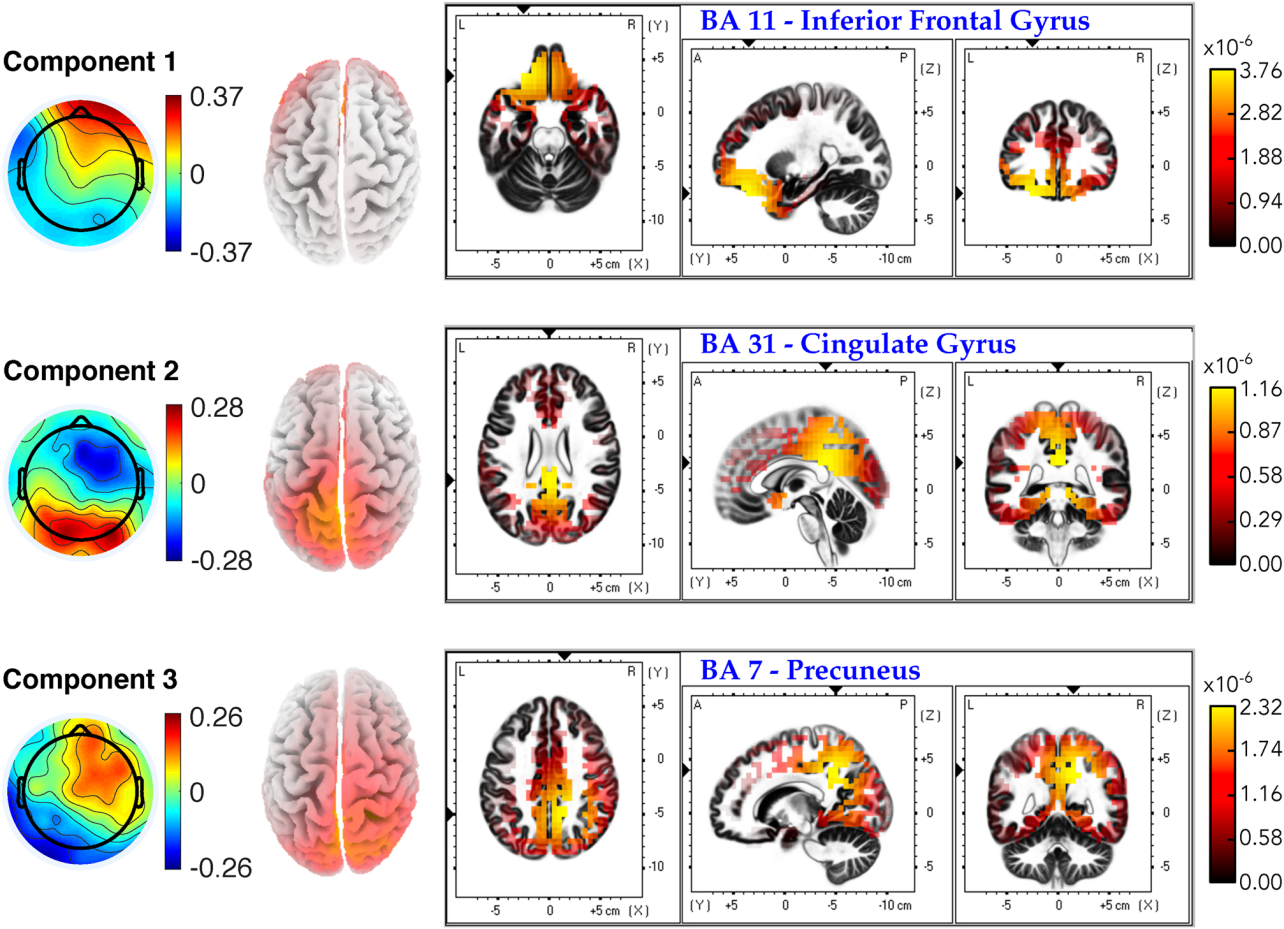
Neural source localization results for the first three ITC components. The scalp projections are shown in the leftmost column for respective components. The second column presents the estimated source distributions (top view). The remaining plots exhibit three orthogonal slices corresponding to the primary sources of the localization results. Full views of 3D source distributions for each component were depicted in Figure SC.2 of Supplementary Material C.

Similarly, the source localization results from the forward models of the ITC analysis are presented in Fig. 8. For the first ITC component, the source analysis disclosed one primary source in BA 11 (Inferior Frontal Gyrus, Frontal Lobe), which is involved in thought and cognitive functions. The second ITC component resulted in one primary source from the BA 31 (Cingulate Gyrus, Limbic Lobe) associated with emotion. Finally, the localization results of the third ITC component revealed one primary source in BA 7 (Precuneus, Parietal Lobe). BA 7 is believed to be associated with visual perception.

## Discussion

In the present study, we designed a decision-making task based on the principle of NP with two difficulty levels. In such a complex business context, decision-making was influenced by external and internal factors^3^, such as provided information, personal experiences, or computational capacity. Neural responses underlying decision-making in this scenario were particularly complicated and altered throughout different experimental periods and trials. Thus, we adopted the CorrCA method^38,39^ to identify linear combinations of EEG channels that maximized the correlation across multiple subjects or trials. Specifically, we made three hypotheses: (1) the attention state of the subjects modulates correlations in EEG signals across subjects and trials; (2) the engagement or newsvendor-decision state of subjects modulates and reduces the alpha and beta-band power; and (3) the neural activities during the NP process involves key cortical regions responsible for cognition and emotion. To test these hypotheses, we first estimated ISC and ITC of the first three components for different task stages under both LM and HM tasks. Then, each spectral power of the three CorrCA-derived projection components was also computed to investigate the power alterations induced by the task difficulty. Finally, eLORETA^42,43^ was employed to translate the ISC and ITC forward models into source distributions of underlying cortical activity. This final step revealed possible cortical sources while subjects were making decisions through the NP-based task.

### Correlations in EEG signals across multiple subjects or multiple trials are modulated by the engagement or attention state

In order to investigate the correlation in EEG signals across multiple subjects and multiple trials, we defined two types of aggregated matrices: (1) trial-aggregated matrix by concatenating multi-trial data along the temporal axis and (2) subject-aggregated matrix by concatenating multi-subject data along the temporal axis. The CorrCA method was then applied to search for the linear combinations of EEG channels that maximize the correlation among subjects or trials. The ISCs and ITCs of the first three components were computed for different experimental periods of both LM and HM tasks. Both ISC and ITC of the first CorrCA component showed significant differences between LM and HM tasks during the DCS period. Since the effort and concentration requirements during the DCS period were substantially distinctive between LM and HM tasks, the results of ISC and ITC confirmed the modulation of the engagement state of subjects on the correlations in EEG signals across multiple subjects or trials.

We observed different trends in the ITC results of the LM and HM groups over different experimental periods compared with those from the ISC analysis. Such a difference can be explained as follows. For the case of HM, since the player can easily earn the profit without having to think carefully during the decision-making period, the subjects’ concentration at this stage decreased more and more throughout multiple trials, which explains why the ITC of the HM task during the DCS period had a weaker ITC value. For the HM group, the highest ITC occurred during the FB period. This observation indicated that subjects were continuously interested in the profits they gained during the game, leading to a higher correlation in the neural signals of the FB period over multiple trials than those during the other periods. For the LM group, because participants always had to think carefully to make the most profitable decisions, the ITC of the LM task during the DCS stage was much higher than that of the HM task. However, although the ITCs of the DCS and FB periods of the LM tasks were higher than those of the resting periods, the differences were not significant as in the ISC case. This observation implies that the correlation in neural responses among trials was weaker than that among multiple subjects.

Existing studies have employed the CorrCA method to investigate the alteration of ISC while subjects were watching movie excerpts^38,48^ or television episodes and commercials^39,49,50^. A more recent study assessed neural correlation during music listening using the same method^51^. ISC extracted from the EEG signals has been demonstrated to reflect various cognitive and behavioral states of subjects, including audience appreciations^38,39^, memory retrieval^48^, or top-down attention^49^. Unlike the mentioned studies that used natural audiovisual stimuli to investigate the ISC of EEG responses, we adopted the NP-based decision-making task as a semi-natural stimulus. In this case, subjects had to face a variety of cognitive and emotional states from the decision-making stage to the feedback stage. Interestingly, CorrCA demonstrated that the challenge levels of decision making (LM vs. HM) modulated and correlated the attentional state of the neural activity of subjects, as represented by the ISC or ITC. The harder the NP was, the more correlated subjects or task trials were.

### EEG power of alpha and delta frequency bands is modulated by the task difficulty

Through the CorrCA method, we were able to identify linear combinations of EEG channels that maximize the correlation across multiple subjects or trials. The CorrCA projection components were then computed by spatially weighting EEG data as spatial filters. We calculated frequency-specific powers of the CorrCA projection components for four experimental periods to investigate further the modulation of the decision difficulty on the spectral power. Statistical analysis revealed significant differences in normalized alpha and beta powers of the three CorrCA components among four task periods. Specifically, the normalized powers during DCS and FB periods were significantly decreased compared to the resting period (i.e., R1). As expected, both DCS and FB periods would require more attention from subjects. Thus, our results demonstrated synchrony reduction of the alpha and beta rhythms by the engagement state of subjects during either DCS or FB.

Our findings were consistent with existing studies in the literature. Various studies have demonstrated correlations between the alpha power of EEG data and the attentional or arousal state of subjects^18,21,49,52–55^. For instance, alpha power can be used to evaluate subjects’ attentional or emotional status while viewing video advertisements^56^. Other studies revealed a decrease of alpha power when people focused on external stimuli^49,53^ or tasks^18,55^. In^57^, the authors investigated the alpha-power lateralization and found that the relationship between alpha-power lateralization and behavioral performance was more complex and depended on baseline alpha power levels.

Furthermore, the beta oscillation was also believed to be strongly correlated with the engagement state of the human brain in cognitive tasks^58–61^ or emotional state during feedback/reward processing^62–64^. For instance, existing studies have demonstrated a decrease in beta-band power when people made effort to memorize^58,59^ or process provided information^60,61^. Moreover, the beta oscillation was assumed to respond to feedback or reward events. Several studies sought to investigate the alterations of beta-band power induced by the “gain” or “loss” feedback conditions^63,64^. In our beta power results, significant differences were found not only between DCS and recovery periods but also between recovery and FB periods. Our results implied that the beta rhythm was strongly correlated among subjects when they received the feedback of gained or lost profit in their last trial during the FB phase.

### Source analysis suggests cognitive and emotional involvement

We further applied eLORETA, a source localization method, to translate the ISC and ITC forward models into distributions of underlying cortical activity. Although the analysis of the cortical origins for the scalp projections of the maximally correlated components could only suggest possible sources of neural activities^65,66^, it helps to induce hypotheses about the spatial origins of neural activities while performing the NP-based task. For the case of maximizing ISC, eLORETA results suggested possible sources in the frontal (BAs 9 and 10), parietal (BA 19), and occipital (BA 23) lobes. BA 9 corresponds to the dorsolateral prefrontal cortex (DLPFC), while BA 10 belongs to the anterior prefrontal cortex (aPFC)^67,68^. Both BAs 9 and 10 are believed to have a significant role in regulating effortful attention and working memory^47,69,70^. Various studies on decision-making using fMRI revealed the activation of BAs 9 and 10 during the decision-making process^71–73^. Our previous study using the same NP-based task with fNIRS also disclosed the brain activation within the DLPFC and partial orbitofrontal cortex (OFC) joined with the frontal polar area (FPA)^33^.

Two other potential sources found in the ISC analysis were BAs 23 and 19. BA 23 is considered a part of the posterior cingulate cortex (PCC). Some studies found evidence for the involvement of PCC in the control of cognition and working memory^74–77^, while others demonstrated the PCC activation by emotional stimuli^78,79^. In our experiment, subjects had to make a decision on the product quantities they wanted to stock and confront a variety of emotional states while receiving feedback on the profits they gained for each trial. Thus, the neural activation of BA 23 in our scenario is reasonable. The last potential activated area, BA 19, corresponds to the visual association cortex. This area is widely known to be associated with complex processing of visual information^80–83^. Since subjects continuously perceive different information on the computer screen throughout the experiment, the activation of this area is understandable.

For the ITC analysis, the source localization resulted in three potential sources: BAs 11, 31, and 7. BA 11 corresponds to the orbitofrontal cortex (OFC), which has been investigated intensively in various studies relating to decision-making and emotional processing^84–87^. For instance, Bechara et al.^84^ assessed the role of OFC in decision-making in a gambling task. Rolls and Grabenhorst^85^ focused on the activation of OFC in emotional processing and decision-making with a variety of rewarding stimuli. Recently, Pelletier and Fellows^87^ reviewed different studies regarding the contribution of OFC to value-based decision-making. Our recent study combining the NP-based task and fNIRS^33^ also revealed the activation of OFC during the decision-making process.

The second possible source of the ITC analysis was BA 31, belonging to the PCC. Interestingly, BA 31 and BA 23, another potential source found in the ISC analysis, are both subregions of the PCC^88^. Although there are some arguments about the PCC’s precise functions due to its specific location that is highly connected with other subcortical regions, some evidence demonstrated that PCC is involved in working memory or computational processes^76,77,88,89^. Finally, the last ITC potential source, BA 7, is a part of the somatosensory association cortex (SAC). The activation of BA 7 may be related to the control of finger movement. Also, BA 7 is believed to be responsive to visual and somatosensory input^90–92^.

### Novel analyses, novel findings, and support for our hypotheses

We applied CorrCA as a novel analysis method to our specific EEG data acquired under the newsvendor decision-making at two difficulty levels. Two data structures were generated to analyze/maximize the inter-subject and inter-trial correlation (ISC and ITC, respectively). We further investigated the normalized power of spatially filtered EEG signals, namely, CorrCA projection components, obtained from both ISC and ITC analysis. Finally, the forward models obtained by the CorrCA method, which represent the scalp projections of the brain activities by the maximally correlated components, were further translated into distributions of underlying cortical activity using eLORETA.

The novel and significant findings of this study were the three supported/proven hypotheses, namely, (1) the attention state of the subjects modulated correlations in EEG signals across subjects and trials at two difficulty levels of the NP; (2) the engagement or newsvendor-decision state of subjects across different task periods modulated and reduced the alpha and beta-band power; and (3) the neural activities during the NP process were involved in key cortical regions responsible for cognition and emotion.

### Limitations and future work

First, although subjects were informed to limit their motion before participating in the study, they still needed to type their chosen value of the product quantity during the DCS period for each trial. In addition, subjects could possibly experience a variety of emotional states while receiving feedback on the gained profits. Both situations could cause unpredictable and/or uncontrollable body motions of subjects during data acquisition. Although pre-processing steps were applied to remove motion artifacts, such noise may not be completely removable. Second, the protocol needed human subjects to read and enter numbers on a computer keyboard. Those actions would involve or stimulate the visual and sensitometer cortex, which should be discriminated from the measured EEG signal for NP-derived correlation analysis. Last, 64-channel EEG would not facilitate high-spatial-resolution, source-localization images. To overcome all these limitations, a larger subject pool should be planned, while an improved protocol design with less dependence on hand/finger movements as well as with a more channel EEG system would be ideal to accurately map neural responses of the human brain to NP decision-making task phases. Moreover, further frequency-band-specific analysis of the source distribution of neural activities could be considered to better understand the cortical distribution of the frequency-dependent neural activities. For instance, the broadband EEG signals could be decomposed into sub-frequency-band signals before applying the CorrCA method.

## Conclusion

The present study sought to investigate the neural mechanisms underlying the newsvendor decision-making process with two challenge levels designed to assess the modulation and correlation of the task difficulty on subjects’ decision-making engagement. We employed the CorrCA method to identify combinations of EEG channels that maximized the correlation across subjects or trials. The ISC and ITC values, which were considered the attentional state marker, were computed for different task periods at the two difficulty levels. The frequency-specific powers of the CorrCA-derived projection components were also calculated. Finally, eLORETA was used to translate the forward models obtained by the ISC and ITC analyses to the potential primary sources of the human cortex. Experimental results and statistical analysis revealed strong and significant correlations in EEG signals among multiple subjects and trials during the difficult decision-making (LM) task than the easier (HM) task. Also, the NP decision-making and feedback phases desynchronized the normalized alpha and beta powers of the EEG, reflecting the engagement state of subjects. Source localization results also revealed several cortical/brain areas during the decision-making process, including DLPFC, aPFC, OFC, PCC, and SAC. These potential sources of neural activities were consistent with results presented in previous studies on decision-making, especially the decision-making in the business context.

## Supplementary Information

Supplementary Information.

## Data Availability

The data presented in this study are available on request from the corresponding author–because we have not set up a public archive platform for data sharing.
