# Supplementary material for: Mapping and understanding of correlated electroencephalogram (EEG) responses to the newsvendor problem
Source: Sci Rep. 2022 Aug 13;12:13800. doi: 10.1038/s41598-022-17970-x (PMC9376113; doi:10.1038/s41598-022-17970-x)
Supplement: Supplementary file 1 — Supplementary Information. [file 41598_2022_17970_MOESM1_ESM.pdf]

Mapping and Understanding of Correlated Electroencephalogram (EEG) Responses to the Newsvendor Problem

Nghi Cong Dung Truong[1], Xinlong Wang[1], Hashini Wanniarachchi[1], Yan Lang[2], Sridhar Nerur[2], Kay-Yut Chen[2], and Hanli Liu[1,*]

[1]Department of Bioengineering, University of Texas at Arlington, 500 UTA Blvd, Arlington, TX 76019, USA
[2]Information Systems and Operations Management, University of Texas at Arlington, 701 S. Nedderman Drive, Arlington, TX 76019, USA
*hanli@uta.edu

Supplementary Material A

Figure SA.1 summarizes the key steps of four main procedure for EEG data analysis, including (A) EEG preprocessing, (B) Quantification of neural correlation across multiple subjects or trials, (C) Quantification of normalized power of CorrCA projection components, and (D) Source localization of correlated neural activity.

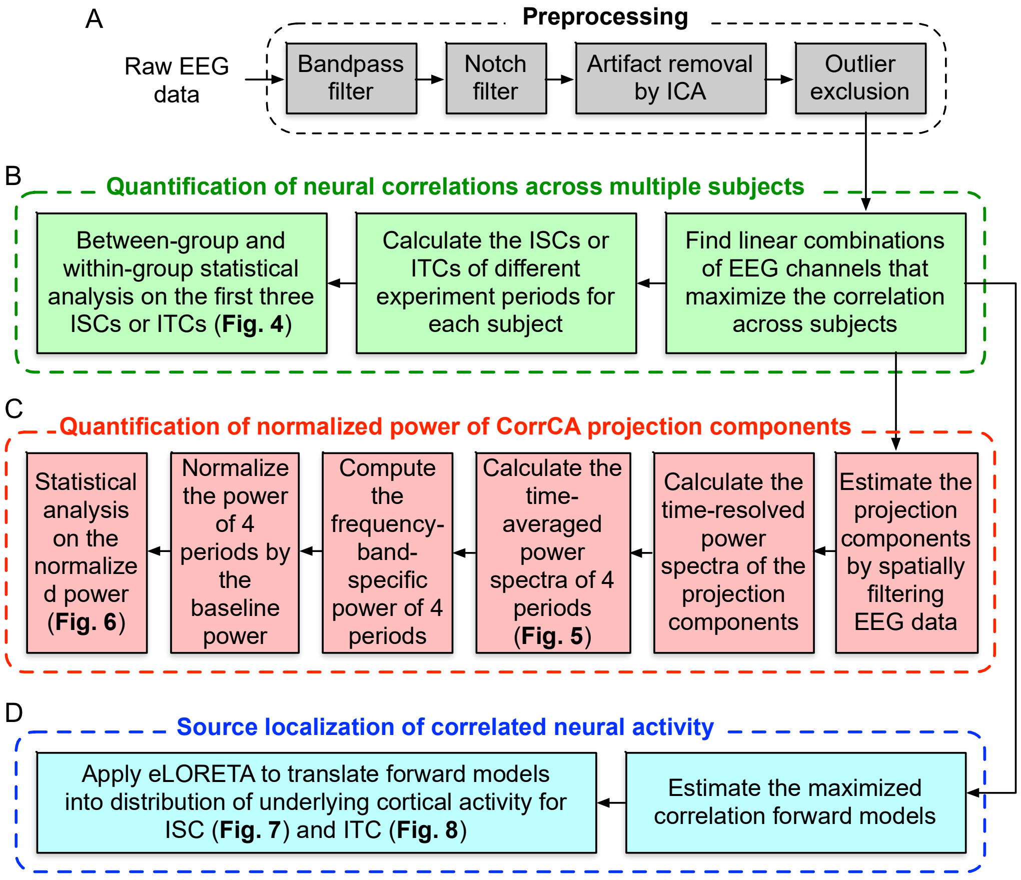

Figure SA.1. Flowchart for EEG data analysis. **(A)** EEG data preprocessing procedure. **(B)** Processing procedure for quantifying neural correlations across multiple subjects or trials. **(C)** Steps for investigating of normalized power of CorrCA projection components. **(D)** Steps for source localization of correlated neural activities.

Supplementary Material B

Tables SB.1 and SB.2 summarize the p-values obtained by the Shapiro-Wilk test of normality of each group for the ISC/ITC and the power data, respectively. Meanwhile, Tables SB.3 and SB.4 depict the p-values of the Levene or Bartlett test of homoscedasticity of the ISC/ITC and the power data, respectively. For all cases, no p-value was less than 0.05. Thus, at the 5% significance level, the data did not provide sufficient evidence to conclude that the data is not from a population with a normal distribution, and the variances from different groups were not significantly different.

Table SB.1 Summary of p-values obtained by the Shapiro-Wilk test of normality of each group for the ISC and ITC data

		**DCS**		**R1**		**FB**		**R2**	
		**LM**	**HM**	**LM**	**HM**	**LM**	**HM**	**LM**	**HM**
**ISC**	Component 1	0.39	0.9	0.56	0.3	0.54	0.23	0.71	0.58
	Component 2	0.29	0.74	0.89	0.31	0.16	0.43	0.24	0.29
	Component 3	0.65	0.07	0.61	0.28	0.19	0.85	0.06	0.36
**ITC**	Component 1	0.08	0.07	0.75	0.3	0.08	0.54	0.52	0.91
	Component 2	0.35	0.87	0.82	0.95	0.85	0.77	0.11	0.77
	Component 3	0.82	0.41	0.12	0.94	0.92	0.28	0.23	0.76

Table SB.2 Summary of p-values obtained by the Shapiro-Wilk test of normality of each group for the power data

		**Alpha**				**Beta**			
		**DCS**	**R1**	**FB**	**R2**	**DCS**	**R1**	**FB**	**R2**
**ISC**	Component 1	0.07	0.21	0.1	0.26	0.3	0.92	0.43	0.91
	Component 2	0.8	0.56	0.93	0.32	0.6	0.59	0.3	0.94
	Component 3	0.35	0.98	0.93	0.47	0.43	0.63	0.36	0.29
**ITC**	Component 1	0.75	0.82	0.94	0.88	0.66	0.67	0.63	0.06
	Component 2	0.64	0.13	0.18	0.31	0.76	0.53	0.08	0.51
	Component 3	0.6	0.42	0.64	0.62	0.2	0.91	0.75	0.8

Table SB.3 Summary of p-values obtained by the Levene's test for homogeneity of variance across groups for the ISC and ITC data

**ISC**			**ITC**		
Component 1	Component 2	Component 3	Component 1	Component 2	Component 3
0.12	0.4	0.69	0.11	0.39	0.7

Table SB.4 Summary of p-values obtained by the Bartlett test for homogeneity of variance across groups for the power data

	ISC		ITC	
	Alpha	Beta	Alpha	Beta
Component 1	0.61	0.48	0.73	0.33
Component 2	0.75	0.27	0.79	0.14
Component 3	0.96	0.14	0.96	0.77

Supplementary Material C

Figures SC.1 and SC.2 depict the 3D source distributions of the underlying cortical density obtained from the ISC and ITC forward models. The primary sources belong to the lobe noted on the top of each component's result.

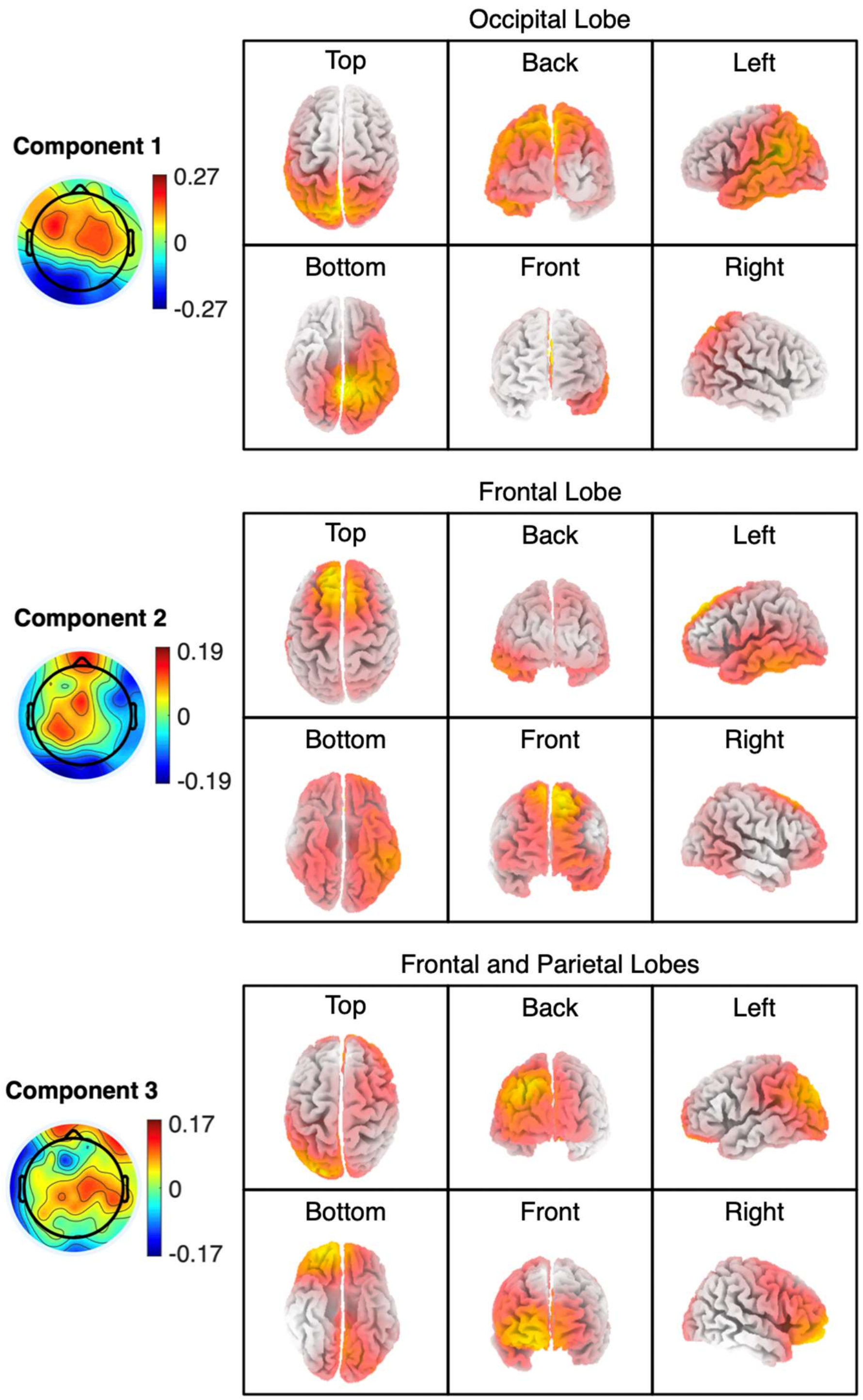

Figure SC.1. 3D source distributions of the underlying cortical density obtained from the ISC forward models

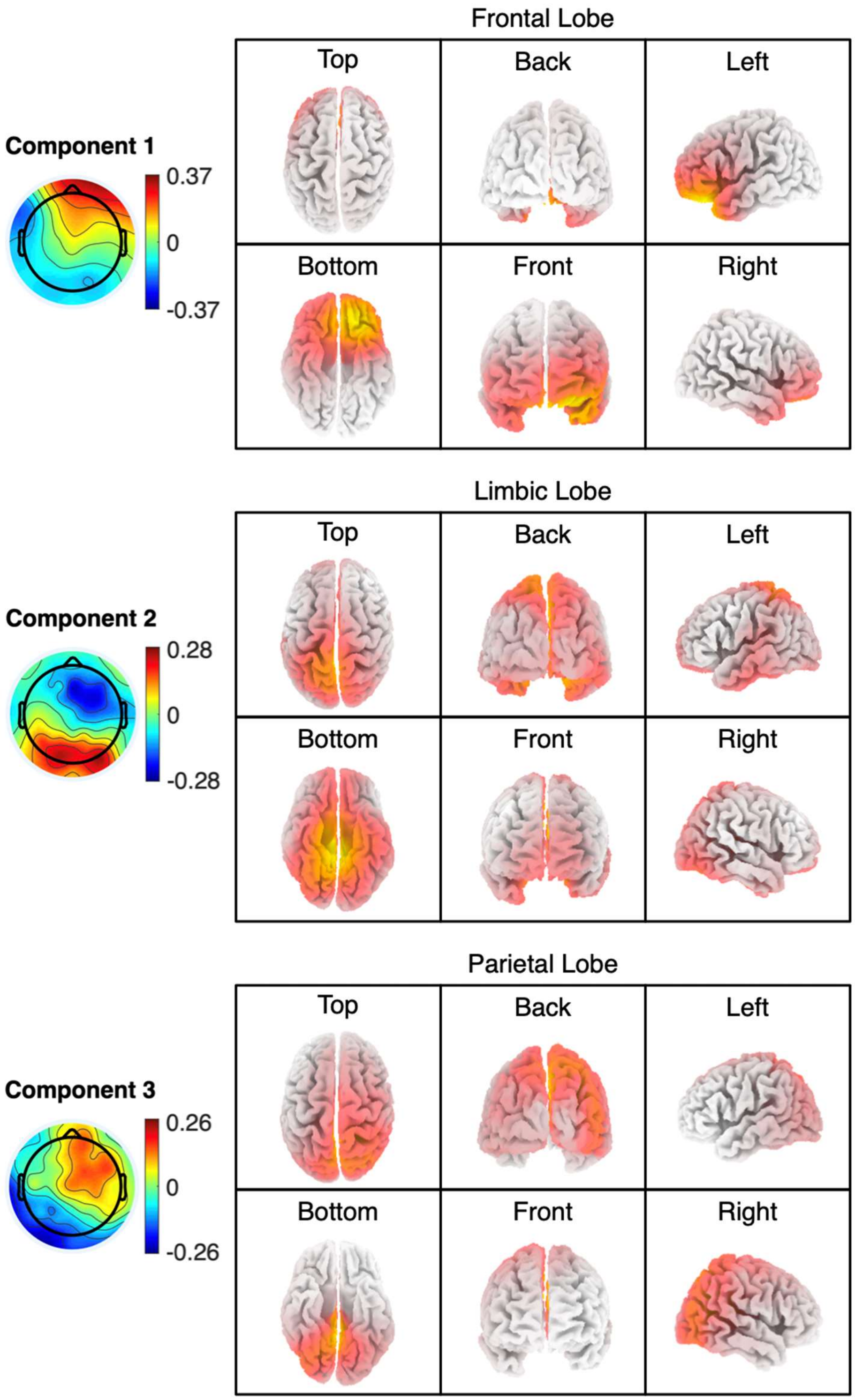

Figure SC.2. 3D source distributions of the underlying cortical density obtained from the ITC forward models
